# Same site recurrence following standardized high ligation and stripping of the great saphenous vein – 5‐year results of the LaVaCro trial

**DOI:** 10.1111/ddg.70012

**Published:** 2026-03-14

**Authors:** Thomas Hummel, Georgios Papapostolou, Margit Broermann, Christoph Dresler, Ingo Flessenkämper, Michael Jünger, Dietmar Stenger, Dominic Mühlberger, Yazad Sidhwa, Thomas Falkenstein, Markus Stücker, Achim Mumme

**Affiliations:** ^1^ Interdisciplinary vein center of the dermatological and vascular surgery clinics, Catholic Hospital of the Ruhr University Bochum Bochum Germany; ^2^ Department of Phlebology Artemed Fachklinik Munich Germany; ^3^ Vein Center Northeim Northeim Germany; ^4^ Department of vascular surgery HELIOS Klinikum Emil von Behring Berlin Germany; ^5^ Department of Dermatology University Hospital Greifswald Greifswald Germany; ^6^ Vein Center Saarlouis Saarlouis Germany

**Keywords:** Great saphenous vein, groin recurrence, high ligation, saphenofemoral junction, varicose veins

## Abstract

**Background:**

Same‐site recurrence at the sapheno‐femoral junction (SFJ) takes up a large amount of the daily practice of phlebologists. Follow‐up data for high‐ligation and stripping (HL/S) of the great saphenous vein (GSV) are quite heterogenous and incoherent. This prospective multicentric study was designed to elucidate the outcome after standardized HL/S with special focus on same site groin recurrences.

**Patients and Methods:**

A standardized HL/S of the GSV, including closure of the cribriform fascia, was performed. The primary endpoint was same‐site groin recurrence, assessed by clinical examination and duplex ultrasonography. Secondary endpoints were recurrences at different sites.

**Results:**

405 patients (519 legs) from six German vein centers were included. 225 patients (287 legs, 55.56%) were available for the five‐year follow up examination. 21 legs (7.32%) suffered from clinically apparent varicosis. Same site groin recurrences were detected in five (1.74%) cases, of which only two were clinically relevant. Most clinically apparent varicose veins were caused by perforator insufficiencies.

**Conclusions:**

The standardized technique of HL/S including ligation of small tributaries of the common femoral vein near the SFJ as well as the closure of the cribriform fascia as an anatomical barrier shows excellent results after five years.

## INTRODUCTION

Saphenofemoral recurrences after crossectomy and stripping of the great saphenous vein (GSV) have substantial socio‐medical and economic relevance.´According to a study by Mumme et al., the annual costs for eliminating recurrences amount to more than 50 million euros per year.^1^ Based on the studies currently available, it is difficult to understand the reality of phlebological care for recurrences in general.

Moreover, groin recurrences are particularly relevant from a hemodynamic point of view. Due to the anatomically high location of the insufficiency point, potentially higher venous pressures can be expected in lower varices than in insufficiencies located more distally. This could lead to an increased number of symptomatic recurrences in the future. For example, Schul et al. demonstrated that side‐branch varicosis is associated with morbidity comparable to that of truncal insufficiency when the level of the reflux origin is anatomically identical.^2^ In the German groin recurrence study, the cause of groin recurrence was determined histopathologically in 427 patients.^3^, ^4^ In 65.3% of cases, the most common cause of recurrence was reflux originating from a residual stump of the GSV that had not been completely removed during the initial operation at the level of the deep vein. However, 16% of recurrences were attributable to neovascularization, resulting in a new connection between the deep and superficial venous systems at the level of the saphenofemoral junction (SFJ). The frequency of saphenofemoral recurrences after a technically correct crossectomy has not yet been clearly established. Assessment of the recurrence rate is hampered by the long latency until the clinical manifestations of recurrence become apparent. According to the results of the German groin recurrence study, it takes 7 to 8 years for saphenofemoral recurrences to become clinically apparent.^3^, ^4^ However, clinical recurrences are preceded by a pathological reflux from the SFJ, which can be detected by duplex sonography, so that a newly occurring sapheno‐femoral reflux can serve as a surrogate parameter for the development of a clinical recurrence.^5^ In several randomized studies assessing the long‐term outcomes of different treatment options for truncal insufficiency of the GSV, duplex ultrasonographic detection of pathological reflux at the saphenofemoral junction was selected as the primary endpoint. A number of studies with a 5‐year follow‐up have now been published.^6^, ^7^, ^8^, ^9^, ^10^, ^11^, ^12^, ^13^, ^14^, ^15^, ^16^, ^17^ In these studies, the recurrence rates varied from 3.8% to 36%. Differences in surgical technique may account for the wide variability in study results, complicating their interpretation.

The aim of our study was to obtain information about the recurrence rates after a technically correct crossectomy in a large group using a strictly standardized surgical technique. To verify this objective, we initiated a prospective observational multicenter trial called LaVaCro (*La*ngzeitergebnisse der *Va*rizenoperation mit *Cro*ssektomie und Stripping der Vena saphena magna).^18^ This analysis presents the 5‐year study results.

## PATIENTS AND METHODS

Eligibility of participating vein centers required a high surgical volume, defined as at least 500 high ligation and stripping procedures (HL/S) of the GSV per year, to ensure adequate surgeon training and expertise. In addition, inclusion of a center in the long‐term outcome analysis required a minimum 5‐year follow‐up rate of 30% to minimize potential bias resulting from high loss to follow‐up or center attrition over time.

The procedures were performed in a standardized manner as previously described.^18^ The standardized procedure included the preparation of the GSV, the SFJ and the surrounding part of the common femoral vein (0.5 cm above and below the SFJ) via a small groin incision. All small branches of the superficial inguinal venous confluence, as well as tributaries directly entering the common femoral vein, were ligated. The GSV was ligated at its confluence with the common femoral vein using a nonresorbable monofilament suture (high ligation). Bipolar coagulation of the exposed endothelium at the ligated stump was additionally performed to reduce neovascularization. Another basic principle of the procedure was the closure of the fossa ovalis with a seam of the cribriform fascia in order to create a natural barrier between the superficial and deep compartments according to Schnyder et al.^19^ Subsequently, invaginated stripping of the GSV was performed, followed by groin wound closure. In addition, mini‐phlebectomies of the thigh and lower leg were carried out to address varicose side branches.

Patients with symptomatic insufficient GSV were included in the study during the clinical routine. According to the classification of Hach,^20^ the reflux in the GSV was divided into four stages (Hach 1: reflux limited to the SFJ, Hach 2: reflux from the SFJ to the thigh, Hach 3: reflux from the SFJ to the calf and Hach 4: reflux from the SFJ to the ankle). Only patients with Hach stage 2–4 were included in the study. In addition, the clinical (C) stage of the CEAP classification was documented.^21^ The study methodology has been reported in detail as previously described.^18^ Patients with a reflux limited to the SFJ (so‐called Hach 1) or missing consent were excluded from the study.

After 5 years, patients were invited for re‐examination. Duplex ultrasonography assessed the following veins: the common femoral vein, with particular focus on the former saphenofemoral junction, the femoral vein, deep femoral vein, popliteal vein, anterior and posterior tibial veins, peroneal veins, and perforator veins of the thigh and calf. The frequency of same‐site groin recurrence after HL/S of the GSV was the primary endpoint of the analysis. Secondary endpoints included other clinically relevant recurrence rates, duplex‐detected recurrence at the thigh and lower leg, and treatment‐related complications or adverse events.

Same site recurrence was defined as a pathological reflux originating from the common femoral vein to the superficial vein system. A reflux longer than 0.5 seconds was considered to be pathological.^22^ Venous reflux without connection to the common femoral vein was not regarded as a same site recurrence. Same‐site recurrences were considered clinically and hemodynamically relevant if the diameter of a single insufficient vein or the combined diameter of all insufficient veins exceeded 5 mm. Furthermore, the CEAP classification was documented, and a duplex ultrasonographic examination of the groin and leg was performed at follow‐up to assess venous status of the superficial and deep compartments and to identify new pathological reflux. Clinically relevant, manifest varicose vein recurrence was evaluated using the Recurrent Varices After Surgery (REVAS) classification.^23^, ^24^

The study was conducted in accordance with the Declaration of Helsinki. The study protocol was approved by the ethics committee of the medical faculty of the Ruhr University of Bochum (Registry No.: 3690‐10). All patients provided written informed consent, and data collection was fully anonymized. Statistical analysis was performed by Microsoft Excel 2010 (descriptive statistics and students t‐test; data are presented as frequency distributions and percentages) and p < 0.05 was regarded significant.

## RESULTS

The LaVaCro‐study originally started with twelve German vein centers.^18^ However, six centers could not achieve the required follow up rate of 30%. Therefore, only six of the participating centers could be included in the evaluation of the long‐term results to avoid major study bias. In the remaining six German vein centers that met the study requirements, a total of 405 consecutive patients (519 legs) could be included in the study. The mean age of the patients was 47.73 years (SD 10.05). Overall, 258 patients (63.70%) were female and 146 (36.05%) were male. The mean preoperative diameter of the great saphenous vein measured by duplex ultrasonography at the saphenofemoral junction was 9.59 mm (SD 2.99). Reflux extending to the calf or ankle, corresponding to Hach stages III and IV, was present in 87.86% of patients. Furthermore, 95% of patients were classified as CEAP clinical class C2 or higher. Further baseline patient characteristics are presented in Table 1.

**TABLE 1:** Baseline demographic, clinical, and treatment data.

Total	n = 405
Average age in years (SD)	47.73 (± 10.05)
Male (%)	146 (36.05)
Female (%)	258 (63.70)
BMI (kg/m^2^) (SD)	27.39 (± 5.81)
Treatment	n = 519
Right leg (%)	257 (49.52)
Left leg (%)	262 (50.48)
Hach classification	
Stage I (%)	0 (0)
Stage II (%)	63 (12.14)
Stage III (%)	287 (55.30)
Stage IV (%)	169 (32.56)
CEAP classification	
C 0 (%)	0 (0)
C 1 (%)	23 (4.43)
C 2 (%)	363 (69.94)
C 3 (%)	100 (19.27)
C 4 (%)	27 (5.20)
C 5 (%)	1 (0.19)
C 6 (%)	5 (0.96)

*Abbr*.: BMI, body mass index; CEAP, Clinical–Etiological–Anatomical–Pathophysiological; SD, standard deviation

Data presented are numbers, mean ± SD or percentage.

The overall complication rate after surgical treatment was 5.39% (n = 28). Hypoesthesia occurred in 22 patients (4.24%), including two cases in the groin and 20 at side‐branch incision sites created during mini‐phlebectomies. Four patients (0.77%) developed hematomas requiring surgical evacuation (two in the groin and two in the lower leg), and two patients (0.39%) presented with lymphatic cysts managed conservatively (one in the groin and one in the thigh).

On the first postoperative day, routine duplex ultrasonography was performed. No residual stump or deep vein thrombosis was detected. Superficial thrombophlebitis of side branches was observed in four legs (0.77%) in the thigh and in three legs (0.58%) in the lower leg. Venous reflux in the superficial venous system with a duration exceeding 0.5 seconds was detected in twelve cases (2.31%) during this early postoperative assessment, including one case (0.19%) in the thigh and eleven cases (2.12%) in the lower leg. Treatment data, complication rates, and postoperative sonographic outcomes are presented in Table 2.

**TABLE 2:** Treatment data, complications, and postoperative sonographic findings.

Total	n = 519
Ambulatory therapy (%)	67 (12.90)
In‐patient treatment (%)	452 (87.09)
Single‐shot Heparin (%)	508 (97.88)
Continued Heparin therapy (%)	15 (2.89)
Single‐shot antibiotic therapy (%)	273 (52.60)
Operative complications	
Motor nerve damage (%)	0
Sensory nerve damage (%)	22 (4.24)
Hematoma (requiring surgery) (%)	4 (0.77)
Lymphatic cyst (%)	2 (0.39)
Postoperative sonographic outcome	
Deep venous thrombosis (%)	0
Residual GSV stump visible (%)	0
Superficial thrombophlebitis of the thigh (%)	4 (0.77)
Superficial thrombophlebitis of the lower leg (%)	3 (0.58)
Venous reflux (thigh) (%)	1 (0.19)
Venous reflux (lower leg) (%)	11 (2.12)

Data presented are numbers and percentages.

After 5 years, 225 patients (287 legs) were available for follow‐up examination, corresponding to a follow‐up rate of 55.56% for patients and 55.30% for legs.

Clinically apparent varicose vein recurrence was observed in 21 of the 287 treated legs (7.32%), prompting evaluation according to the REVAS protocol. In these cases, no topographical association with the groin was identified. One leg (0.35%) exhibited cross‐groin insufficiency originating from pelvic veins, and popliteal perforator insufficiency was detected in two legs (0.70%). The majority of clinically apparent recurrences were attributable to perforator insufficiencies of the thigh and lower extremity.

The results of duplex ultrasonography differ from those of the REVAS classification, as all reflux detected on duplex imaging is recorded, including clinically inapparent reflux. Same‐site recurrent groin reflux was detected by duplex ultrasonography in five cases (1.74%). Only one case (0.35%) exhibited a hemodynamically relevant insufficient vein diameter of 7 mm. The diameters of the remaining four groin‐insufficient veins were < 3.5 mm and were therefore classified as not hemodynamically relevant. Thigh reflux was detected in 22 legs (7.67%), with a mean maximum vein diameter of 3.18 mm (SD 1.67). In addition, 28 insufficient superficial veins were identified in the lower extremity, with a mean diameter of 2.80 mm (SD 1.43). The 5‐year results are summarized in Table 3.

**TABLE 3:** Five‐year results.

Total	
Follow up patients (%) (initial n = 405)	225 (55.56)
Follow up legs (%) (initial n = 519)	287 (55.30)
Clinically apparent varicosis (REVAS) (%)	21 (7.32)
Groin varicosis	0
Thigh varicosis (%)	1 (0.35)
Thigh and lower leg varicosis (%)	10 (3.48)
Lower leg varicosis (%)	8 (2.79)
Popliteal varicosis (%)	2 (0.70)
Sonographic outcome	
Reflux at the sapheno‐femoral junction (%)	5 (1.74)
Sum of venous diameter at the groin in mm (SD)	4,00 (± 1.70)
Reflux at the thigh (%)	22 (7.67)
Max. vein diameter (thigh) in mm (SD)	3,18 (± 1.67)
Reflux at the lower leg (%)	28 (9.76)
Max. vein diameter (lower leg) in mm (SD)	2,80 (± 1.43)
CEAP classification	
C 0 (%)	85 (29.62)
C 1 (%)	133 (46.34)
C 2 (%)	60 (20.91)
C3 (%)	9 (3.14)
C 4 (%)	0
C 5 (%)	0
C 6 (%)	0

*Abbr*.: CEAP, Clinical–Etiological–Anatomical–Pathophysiological; REVAS, recurrent varicose veins after surgery; SD, standard deviation

Data presented are numbers, mean ± SD or percentage.

Student's t‐test demonstrated a significant improvement in the CEAP clinical (C) stage at 5‐year follow‐up compared with preoperative findings (p < 0.001). The distribution of C stages before surgery and at 5 years postoperatively is shown in Figure 1.

**FIGURE 1:**
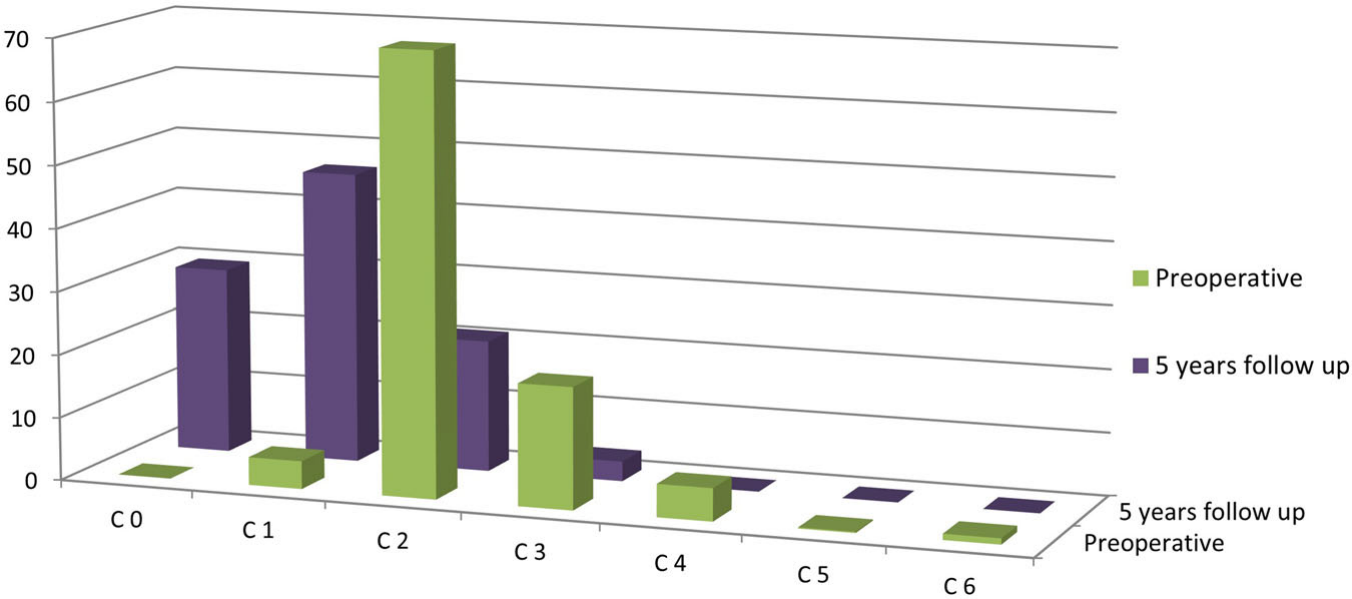
CEAP classification: percentage distribution of C stages preoperatively and after 5 years.

## DISCUSSION

In this prospective multicenter study, the same‐site duplex‐detected recurrence rate at the saphenofemoral junction after standardized high ligation was 1.74% at 5‐year follow‐up. Only one case (0.35%) showed hemodynamically relevant insufficiency, defined as a cumulative vein diameter > 5 mm. Although the present study was designed to focus on groin recurrence, clinical recurrences were observed in 7.32% of cases at 5‐year follow‐up and were therefore lower than expected. These data contrast with previously published studies, as they demonstrate a lower same site groin recurrence rate. For example, Allegra et al. reported in a prospective study that, after flush ligation of the GSV, the same‐site recurrence rate at the SFJ was 13% (109/862) as detected by duplex sonography at 5‐year follow‐up.^15^ Other prospective studies showed recurrence rates at SFJ of 11.7% after 6 years or 7% after 5 years.^13^, ^14^ However, it should be noted that the surgical technique used in the studies cited was not described in sufficient detail and that a partially heterogeneous patient group was included. A direct comparison with the data collected in our study is therefore difficult. The most detailed description of the surgical technique was provided in the RELACS study by Rass et al.^16^ In this study, crossectomy was performed with transection of all groin tributaries and flush ligation of the saphenofemoral junction using a nonresorbable 0‐0 Ethibond suture (Ethicon, New Brunswick, NJ, USA). Neoreflux protection was achieved by an invaginating continuous Prolene 4‐0 stump suture (Ethicon), followed by invagination stripping of the great saphenous vein to just below the knee. Although the authors reported higher duplex sonography–detected same‐site recurrence rates at the saphenofemoral junction of 5% at 5‐year follow‐up after HL/S, these rates were higher than those observed in our study.

The lower rate of same‐site recurrences observed in our study may be attributable to the specific surgical technique used for HL/S of the GSV. First, the common femoral vein was exposed over a limited segment, and all directly entering tributaries were carefully dissected.

Anatomical studies have confirmed the presence of at least one vein entering the common femoral vein directly at the level of the SFJ in 57% of cases.^25^ Additionally, we used a monofilament non‐resorbable ligature for the GSV stump and the exposed intima of the stump was destroyed by bipolar coagulation to avoid possible neointimal induction. When comparing the surgical procedures, thermal ablation was not included in the RELACS study cited.^15^ It cannot be stated with certainty whether this contributed to the improved outcome.

There is low evidence that the recurrence rate with non‐resorbable sutures is lower than with absorbable sutures in the SFJ.^26^ Frings et al. reported a two‐third reduction of SFJ recurrence after GSV intima stump exclusion with a burial by an overlying running, synthetic monofilament suture,^27^ compared with an exposed stump and ligature while other authors could not confirm these results.^28^ On the other hand, all involved surgeons in our study were well trained in the atraumatic preparation of the SFJ, with minimalized tissue stress and hematoma to prevent angiogenic stimulus. This might contribute to the good results as well, as the surgeon's qualification may influence the outcome.^29^

Moreover, we believe that the closure of the cribriform fascia is another crucial part to prevent same site recurrences after HL/S of the GSV. This closure of the fossa ovalis may act like an anatomical barrier to divide the deep and superficial vein compartments. This is also a significant difference to the RELACS study protocol described above, in which no closure of the fascia or other barrier measures were performed.^16^ The principle of a prosthetic or anatomical barrier to avoid neovascularization and prevent recurrence was proposed 18 years ago.^30^ Furthermore, De Maeseneer et al. demonstrated that the closure of the cribriform fascia with or without a prosthetic material significantly reduces same site recurrence at the SFJ after HL/S of GSV.^31^, ^32^ Based on these results, the closure of the cribriform fascia was included in this trial as a potential barrier from transfascial insufficiencies.

In addition, the duplex sonographic detection of this neoreflux in untreated tributaries of the SFJ could be of clinical relevance in the future, as recurrent groin reflux at the SFJ detected by duplex sonography is a well‐known surrogate parameter for the development of clinical recurrence in the long term.^5^, ^16^ Therefore, the published short‐ and mid‐term duplex recurrence rates at the SFJ after surgical and endovascular treatments must be observed carefully, as they may allow conclusions about the long‐term clinical outcome for these procedures. Regarding this aspect, nowadays most data are available for endovascular laser ablation (EVLA). For example, in comparable studies with five or more years follow up, EVLA demonstrated significantly more duplex‐detectable same site recurrences at the SFJ.^13^, ^16^, ^33^ Independent of the duplex findings, Rass et al. and Gauw et al. could demonstrate that EVLA showed significantly more clinically relevant same site recurrences at the SFJ compared to HL/S after five years.^14^, ^16^ However, no significant differences were found between the two groups in terms of overall clinical recurrence rates in these studies. According to the data of the German groin recurrence study, clinically apparent same site recurrences appear after an interval of 7 to 8 years.^3^, ^4^ This could indicate that future studies on this topic need longer follow‐up to be able to accurately compare the clinical outcomes of the different therapies. To our knowledge, only one study with 10‐year long‐term results on this topic has been published so far.^34^ In this study, significantly fewer clinically apparent same site recurrences were found originating from the groin after HL/S compared to EVLA. However, the results of this study can only be evaluated with caution due to study limitations.

The low groin recurrence rate in the present study with a technically correct crossectomy after five years confirms the motto formulated by the phlebological pioneers Dodd and Nabatov: “no stump – no recurrence”.^35^, ^36^

This statement may be the basis for the fact that endovenous therapy using the so‐called “endovenous crossectomy” can also achieve a reduction in the recurrence rate, as a recently published randomized controlled study showed.^37^

Patients’ perspectives, expectations, and preferences are important for future service provision and play a decisive role in treatment choice. In a survey of the preferences and expectations of patients regarding the treatment of their varicose veins, Shepard et al. were able to show that more than half of the patients prefer treatment in a single session instead of repeated therapy.^38^ Measures to prevent recurrences are relevant factors in the treatment decision for patients and are worth considering.

### Limitations

Nevertheless, there are several limitations of the present study. A major limitation is that six of the initial twelve vein centers had to be excluded from the evaluation of the long‐term data due to a lack of follow up examinations. There are multiple reasons for this. For example, some of the initial clinical investigators quit or changed in the centers over time. These limited follow up data reflect the difficult study conditions for multi‐center investigations in varicose vein diseases in Germany. One reason for this is that vein therapy in Germany is mainly carried out in non‐university, outpatient settings. Nowadays, these vein centers are subject to strong economic and personnel restrictions, so that the external, surrounding study conditions for the individual centers have changed over time. This may explain the limited number of participating study centers for the five‐year evaluation. Even so, we could reinvestigate 287 legs 5 years after the initial HL/S in total, with a follow up rate of 55,56%. Therefore, it cannot be ruled out that the reduced follow‐up rate led to a distortion of the results. Compared to other studies on this topic, we included higher case numbers and follow‐up rates,^14^, ^15^, ^16^, ^17^, ^19^ so we believe that the results of our investigation can make a significant contribution to the scientific discussion. One further limitation of our study is the lack of a comparative approach or a control group. Regardless, this study was designed as an observational study, to elucidate the results of a standardized HL/S technique with particular attention to same site recurrences at the groin in long term follow up, because many studies deal with historical HL/S results as a comparative group without defining specific operative techniques. The lack of a control group or a missing comparative approach does not prevent a solid interpretation of the results.

## Conclusions

In this study, we could demonstrate that a specific standardized HL/S has a same site recurrence rate of 1.74% after 5 years. These results underline the effectiveness of high ligation in the prevention of groin recurrence. It remains unclear which part of the technique used is crucial for these good results. At any rate, comparative studies with long term follow up are needed to evaluate this standardized high ligation in comparison to other procedures regarding same site recurrence rates.

## FUNDING

This study was initiated and financially supported by the vein surgery working group (VOP‐AG) of the *German Society of Phlebology* (DGP). Financial support has no role in the analysis and interpretation of data.

## CONFLICT O INTEREST STATEMENT

None.
